# Outdoor time, screen time and sleep reported across early childhood: concurrent trajectories and maternal predictors

**DOI:** 10.1186/s12966-022-01386-x

**Published:** 2022-12-29

**Authors:** Katherine L. Downing, Borja del Pozo Cruz, Taren Sanders, Miaobing Zheng, Jill A. Hnatiuk, Jo Salmon, Kylie D. Hesketh

**Affiliations:** 1 Institute for Physical Activity and Nutrition (IPAN), School of Exercise and Nutrition Sciences, Deakin University, Geelong, Australia; 2 Center for Active and Healthy Ageing, Department of Sports Science and Clinical Biomechanics, Faculty of Health Sciences, University of Southern Denmark, Odense, Denmark; 3 Institute for Positive Psychology and Education, Faculty of Health Sciences, Australian Catholic University, Sydney, Australia

**Keywords:** 24-h movement behaviours, Outdoor time, Sedentary behaviour, Sleep, Early childhood, Trajectories, Maternal factors

## Abstract

**Background:**

Understanding the developmental trajectories of outdoor time, screen time and sleep is necessary to inform early interventions that promote healthy behaviours. This study aimed to describe concurrent trajectories of outdoor time, screen time and sleep across the early childhood period and their maternal predictors.

**Methods:**

Data across five time points at child age 4, 9, 19, 42 and 60 months from the INFANT intervention were analysed. Mothers reported their child’s usual outdoor time, screen time and sleep duration, in addition to a range of maternal beliefs, attitudes, expectations and behaviours. Group-based multi-trajectory modelling was used to model concurrent trajectories of children’s behaviours. Multinomial logistic regression models determined the associations of maternal predictors with trajectory groups, adjusting for child sex and baseline age, intervention allocation, and clustering by recruitment.

**Results:**

Of the 542 children recruited, 528 had data for outdoor time, screen time and sleep at one or more time points and were included in trajectory analyses Four trajectories were identified: *‘unstable sleep, increasing outdoor time, low screen’* (~ 22% of sample), *‘high outdoor time, low screen, high sleep’* (~ 24%), *‘high sleep, increasing outdoor time, low screen’* (~ 45%), *‘high screen, increasing outdoor time, high sleep’* (~ 10%). The *‘high sleep, increasing outdoor time, low screen’* group, comprising the largest percentage of the sample, demonstrated the healthiest behaviours. Predictors of group membership included: views of physically active children, screen time knowledge, screen time use, self-efficacy, physical activity optimism, future expectations for children’s physical activity and screen time, perceptions of floor play safety, and maternal physical activity, screen time, and sleep quality.

**Conclusions:**

Four distinct trajectories of outdoor time, screen time and sleep were identified, with the most common (and healthiest) characterized by high levels of sleep. Maternal beliefs, attitudes, expectations and behaviours are important in the development of movement behaviour trajectories across early childhood. Future interventions and public policy may benefit from targeting these factors to support healthy movement behaviours from a young age.

**Supplementary Information:**

The online version contains supplementary material available at 10.1186/s12966-022-01386-x.

## Background

Increased physical activity, reduced sedentary behaviour (e.g., screen time) and optimal sleep duration are independently associated with positive physical, psychosocial and cognitive health outcomes in early childhood (birth through 5 years) [1–3]. Engagement in sufficient amounts of these movement behaviours *in combination*appears to provide the most beneficial outcomes for both children and youth [4, 5], but cross-sectional evidence suggests that most children do not achieve optimal time in all three behaviors [6–8]. Longitudinal evidence across childhood has mostly examined mean change in separate behaviours (e.g., physical activity) over time and ‘tracking’ of these behaviours (i.e., a child’s maintenance of relative rank in a cohort over time). Total time spent in physical activity tends to decline with age while time spent in sedentary behaviour increases [9]. Sleep duration also decreases with age [10]; however, this is likely to be developmentally appropriate (i.e., children need less sleep as they age). In addition, physical activity and sedentary behaviour show moderate to high tracking [11], suggesting that children tend to maintain their physical activity and sedentary behaviour levels compared to peers, even if the total minutes of time in these behaviours within the cohort shifts over time. Conversely, sleep duration has been shown to track poorly [12].

Research regarding how these movement behaviours *concurrently*change over time is limited. Physical activity, sedentary behaviour and sleep are interrelated, in that time spent in one behaviour takes away available time for the remaining behaviours (i.e., time substitution) [13]. Therefore, changes in these behaviours over time are likely to be related. Although longitudinal trajectory analyses are increasingly being used to examine simultaneous changes in young children’s physical activity and screen time [14, 15], to date sleep has been overlooked. Understanding the developmental trajectories of all three movement behaviours concurrently is critical for early identification of those most at risk of developing detrimental behavioural profiles.

A key challenge in determining change over time in early childhood movement behaviours is the difficulty in assessing physical activity in young children. In the first two years of life, infants and toddlers experience a rapid increase in motor control and ability [16], meaning that their physical activity types and levels change and increase dramatically. This is also reflected in the operationalisation of physical activity in current guidelines; for infants not yet mobile, physical activity recommendations suggest 30 min of tummy time spread across the day [17, 18]. For toddlers and preschoolers (aged 1 year and over), the recommendations suggest 180 min in a variety of physical activities [17, 18] (operationalised as 180 min of total physical activity). As such, finding a consistent measure of physical activity across the entire early childhood period is difficult. Outdoor time has been shown to be a useful proxy for physical activity in children as young as 2 years; evidence suggests that 70% of outdoor time is active, with more than 20% of outdoor time spent in higher intensity activity [19]. There is also preliminary evidence that a large percentage (57%) of infants’ and toddlers’ (ranging from 6 weeks to 36 months of age) outdoor time in childcare is spent active [20].

It is also important to determine the characteristics that predict ‘healthy’ vs ‘unhealthy’ trajectories as potential intervention targets. Parents are the biggest influence on young children’s health behaviours, with evidence suggesting that parental beliefs, attitudes and behaviours are important factors associated with young children’s physical activity, screen time and sleep [21, 22]. In particular, parental encouragement and support appear to be important for children’s physical activity [20], while parents own screen time and sleep duration are associated with children’s screen time [20] and sleep duration [22], respectively. To our knowledge, no studies have investigated concurrent trajectories of these movement behaviours in early childhood and the parental characteristics that influence them. This study aimed to: (1) describe concurrent trajectories of outdoor time, screen time and sleep across the early childhood period; and (2) examine maternal predictors of those trajectories.

## Methods

Data were drawn from INFANT (2008–2013), a randomized controlled trial that aimed to prevent obesity and obesity-promoting behaviours (including diet, physical inactivity, and sedentary behaviours) from child ages 4 to 19 months, with follow-up at ages 3.5 and 5 years. The trial has been previously described [23, 24]; details relevant to the current study are described here. Participants were recruited from 14 local government areas randomly selected from all those within a 60 km radius of Deakin University’s Burwood campus, located in Melbourne, Australia. Within participating local government areas, 50% of first-time parents’ groups were randomly approached for participation in the study (*n* = 62 groups). First-time parents’ groups, formed and facilitated by the free, universal Maternal and Child Health service within Victoria, are predominantly attended by mothers (in the present study all participants were mothers). Inclusion criteria was a minimum of eight mothers within a group consenting to participate, or a minimum of six mothers in low socioeconomic areas [25]. Where a group declined to participate or did not meet the inclusion criteria, the next group on the randomly generated list was approached. Deakin University’s Human Research Ethics Committee (EC 175–2007) and the Victorian Government’s Office for Children granted approval to conduct INFANT.

### Measures

Measures were taken at baseline (T1: child age approximately 4 months), mid-intervention (T2: age 9 months), intervention conclusion (T3: age 19 months), and at two post-intervention follow-ups (T4: age 42 months; and T5: age 60 months). Participants in both the intervention and control group were included in the current study, as sleep was not a target in the intervention (and there was no difference in sleep duration between groups), no intervention effect was observed for physical activity, and the intervention effect for screen time was attenuated at follow-up. However, intervention allocation was included as a covariate to adjust for any potential confounding effect.

### Demographic characteristics

At T1, mothers reported their highest level of education (categorized as some high school, completed high school/ trade/ certificate qualification, or university), and the sex and date of birth of their child via questionnaires. Trained researchers measured the child’s length (m) using a calibrated measuring mat (Seca 210, Seca Deutschland, Germany) and weight (kg) using calibrated digital scales (Tania 1582, Tokyo, Japan). Body mass index (BMI) z-scores were calculated according to the World Health Organization age- and sex-specific growth charts [26].

### Predictors

Maternal factors (knowledge, beliefs, attitudes, and expectations) were assessed at T1 through 36 purpose-designed questionnaire items, based on formative work [27, 28]. The items were tested in a separate sample and showed moderate to good test–retest reliability (% agreement = 0.56–0.86). All items were answered on a 4-point Likert scale (0 = strongly disagree/not at all confident to 3 = strongly agree/extremely confident) and were coded for the current study such that a higher score indicated agreement with current evidence and recommendations. As previously described [29], nine factors were generated using exploratory factor analyses: physical activity knowledge (e.g., importance of physical activity for babies’ and toddlers’ health and development); views of physically active children (e.g., active babies are easier to look after); physical activity optimism (e.g., anticipated ease of engaging children in physical activity); self-efficacy for promoting physical activity; future expectations for children’s physical activity and screen time; perceptions of floor play safety (e.g., not concerned about baby hurting themselves if left lying on the floor); screen time knowledge (e.g., perceived detriment of television for young children); screen time use for practical reasons (e.g., using television to keep child occupied); and self-efficacy for limiting screen time. As sleep was not a focus of the intervention, factors relating specifically to sleep were not measured. Factor scores were generated by averaging the item scores within each factor. All factors had good internal reliability (Cronbach’s α = 0.58–0.87).

Mothers’ moderate- to vigorous-intensity physical activity (MVPA) was assessed at T1 using the Active Australia Survey [30] and converted to hours per day. Mothers reported their television viewing time on a usual weekday and a usual weekend day, weighted to average hours per day [31]. Mothers also reported the quality of their own sleep over the past week on a 4-point Likert scale (0 = very bad to 3 = very good), collapsed into ‘bad’ and ‘good’.

### Outcomes

Children’s physical activity was operationalized in the current study as time outdoors, which is often used as a proxy for physical activity in this age group [32–34]. Mothers reported the amount of time their child spent outdoors on an average day at each time point. At T1-T3, screen time was reported as the number of hours and minutes that the child spent watching or in front of the television on a typical day. At T4 and T5, parents reported their child’s total screen time (hours and minutes) on a typical weekday and a typical weekend day, weighted to an average day. Sleep was reported as the usual number of hours and minutes of sleep at night and daytime naps at each time point. Night and daytime sleep were summed to give total sleep duration. All behaviour variables were converted to hours per day.

### Statistical analysis

Using the traj procedure [35, 36] in Stata/SE 16.0 (StataCorp, Texas, USA), group-based multi-trajectories were modelled for outdoor time, screen time and sleep from child age 4 to 60 months. Group-based trajectory modelling (GBTM) is a form of finite mixture model that allows the shape of the trajectories to vary across groups [37]. Trajectory models are fitted to subgroups identified by the data [37], rather than predefined subgroups (e.g., classification based on change between two time points). Multi-trajectory modelling is a form of GBTM, based on semiparametric mixture models and maximum-likelihood, that allows for simultaneous estimation of trajectories for multiple outcomes [36]. First, we estimated and compared joint censored normal models with two to five latent groups. The best model in terms of number of groups and shapes of trajectories (i.e., linear, quadratic, cubic) was determined using the Bayesian Information Criterion [37]. Multinomial logistic regression models were used to determine the associations of each of the predictor variables with the trajectory groups, adjusting for child sex and baseline age, intervention allocation, and clustering by first-time parent group.

### Sensitivity analyses

We conducted sensitivity analyses utilising accelerometer-derived physical activity in place of outdoor time from the later three time points only (i.e., child ages 19, 42 and 60 months). At these three timepoints, children wore ActiGraph™ GT1M accelerometers (Pensacola, FL, USA) on an elasticised belt at the right hip during waking hours for eight consecutive days. Movement counts were recorded in 15-s epochs, with epochs > 25 counts defined as total physical activity (TPA) [38]. Non-wear time, defined as ≥ 20 min of consecutive zero counts, was removed. Children with at least 4 days of ≥ 7.4 h of recorded data were included in analyses [39]. We utilised the same model fit characteristics as the main trajectory analyses to allow for comparison. Multinomial logistic regression models were used to determine the associations of each of the predictor variables with the new trajectory groups, adjusting for child sex and baseline age, intervention allocation, and clustering by first-time parent group.

## Results

**Fig. 1:**
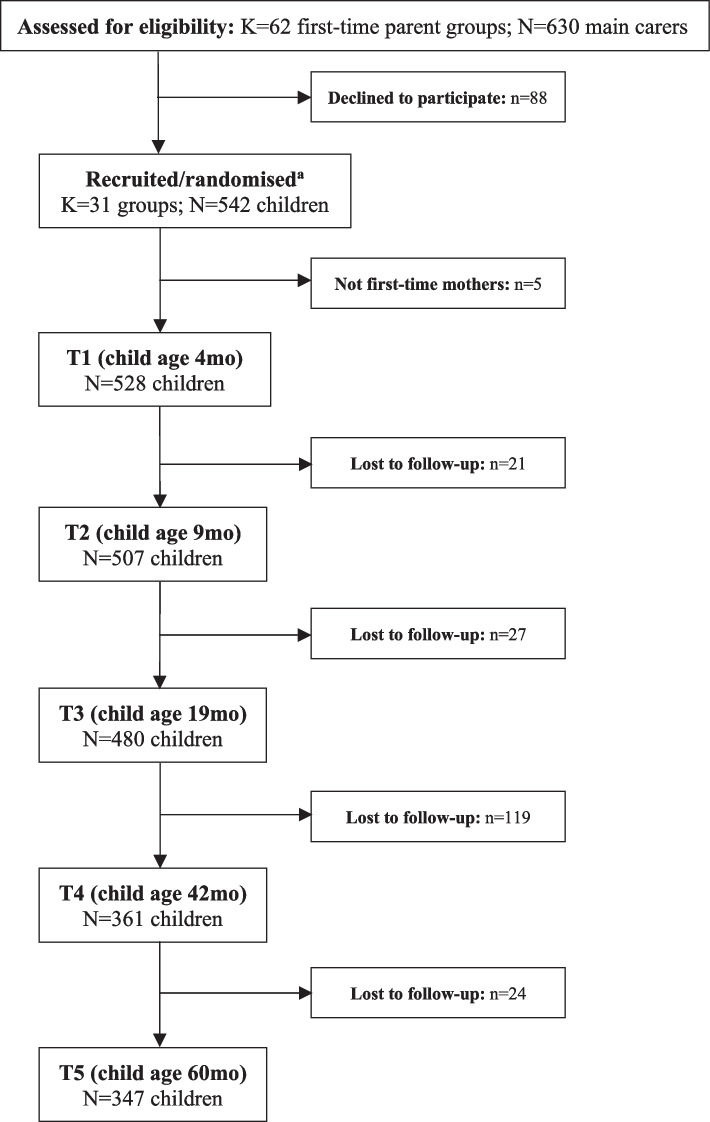
Participant flow chart Note: ^a^ Randomisation (i.e., numbers in intervention/control groups) are not reported as data were treated as cohort data for the present study

Of the 542 children recruited, 528 had data for outdoor time, screen time and sleep at one or more time points and were included in trajectory analyses (Fig. 1), and 468 had complete data for predictors. Baseline participant characteristics are shown in Table 1.

**Table 1:** Participant baseline characteristics

	Mean (SD) *n* = 468
**Child characteristics**	
Sex, female (%)	47.7
Age, months	3.6 (1.0)
Length, m	0.6 (0.03)
Weight, kg	6.2 (0.9)
BMI z-score	-0.5 (1.0)
**Maternal characteristics**	
Highest level of education (%)	
Some high school	20.9
Completed high school/ trade/ certificate	25.6
University	53.4
**Maternal factors**	
PA knowledge *(possible range 0–3)*	2.5 (0.3)
Views of physically active children *(possible range 0–3)*	0.9 (0.5)
PA optimism *(possible range 0–3)*	2.3 (0.4)
Self-efficacy for promoting PA *(possible range 0–3)*	2.5 (0.5)
Future expectations around children’s PA and ST *(possible range 0–3)*	1.7 (0.6)
Floor play concerns *(possible range 0–3)*	1.0 (0.6)
ST knowledge *(possible range 0–3)*	1.4 (0.5)
ST use for practical reasons *(possible range 0–3)*	2.2 (0.5)
Self-efficacy for limiting ST *(possible range 0–3)*	2.0 (0.6)
MVPA (hours/day)	1.2 (0.9)
ST (hours/day)	0.5 (1.9)
Good sleep quality (%)	78.9

Abbreviations: *BMI* Body mass index, *MVPA* Moderate- to vigorous-intensity physical activity, *PA* Physical activity, *SD* Standard deviation, *ST* Screen time

### Concurrent trajectories of movement behaviours

**Fig. 2:**
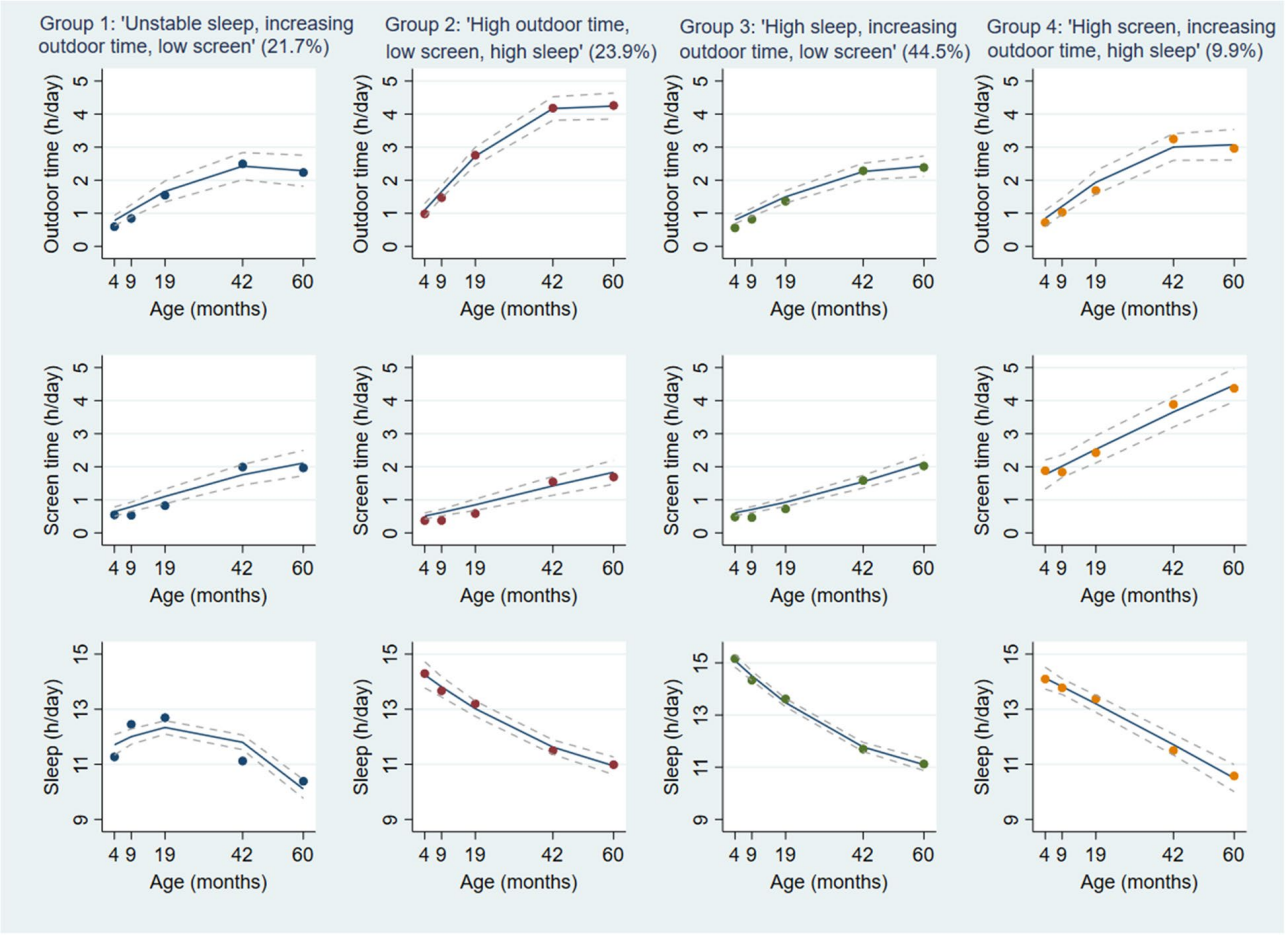
Concurrent trajectories of movement behaviors across early childhood Abbreviation: h, hours

Model fit characteristics are shown in Additional file 1 (Table S1). The best model (i.e., the model with the lowest Bayesian Information Criterion) identified four groups (Fig. 2). Each group showed a distinctive trajectory for one of the three behaviours. Group 1, named *‘unstable sleep, increasing outdoor time, low screen’*, comprised 21.7% of the sample. Group 2 comprised 23.9% of the sample and was named *‘high outdoor time, low screen, high sleep’*. Group 3 comprised the highest proportion of the sample (44.5%) and was named *‘high sleep, increasing outdoor time, low screen’*. Finally, Group 4, named *‘high screen, increasing outdoor time, high sleep’*, comprised the smallest proportion of the sample (9.9%). Additional file 2 (Table S2) shows descriptive characteristics of time spent in the three behaviours for the total sample and the four groups. The differences in outdoor time and screen time between groups increased over time. At age 4 months, outdoor time differed by 30 min per day and screen time by approximately 90 min per day between groups, increasing to approximately 2 and 2.5 h per day, respectively, at 60 months. Conversely, sleep stabilized over time, with the difference between groups decreasing from around 4 h per day at age 4 months to less than 1 h per day at 60 months.

### Maternal predictors of concurrent trajectories of movement behaviours

Associations of maternal factors with trajectory groups are shown in Table 2. Initially, the *‘high outdoor time, low screen, high sleep’* group was used as the referent group, as those showing the ‘optimal’ movement behaviour trajectories (i.e., the healthiest trajectories in terms of guideline compliance across the three behaviours). Views of physically active children (e.g., active babies are easier to look after), screen time knowledge (e.g., perceived detriment of television for young children), screen time use for practical reasons (i.e., parent disagrees with using screens for practical reasons), and self-efficacy for limiting screen time were associated with 43–97% lower odds of children being in any of the other three groups compared to the referent group. Physical activity optimism (e.g., anticipated ease of engaging children in physical activity) and future expectations for children’s physical activity and screen time were associated with decreased odds (44% and 39%, respectively), and perceptions of floor play safety (e.g., not concerned about baby hurting themselves if left lying on the floor) were associated with 173% increased odds, of children being in the *‘unstable sleep, increasing outdoor time, low screen’*. Self-efficacy for promoting physical activity was associated with 57% decreased odds of children being in the *‘high screen, increasing outdoor time, high sleep’* group. In terms of maternal behaviours, maternal MVPA was associated with 23% and 40% decreased odds of children being in the *‘unstable sleep, increasing outdoor time, low screen’* and *‘high sleep, increasing outdoor time, low screen’* groups, respectively, while maternal screen time was associated with 336% increased odds of children being in the *‘high screen, increasing outdoor time, high sleep’* group compared to the referent group.

**Table 2:** Associations of maternal factors with concurrent trajectories of movement behaviours

	OR (95% CI)			
	*High outdoor time, low screen, high sleep* (referent group)	*Unstable sleep, increasing outdoor time, low screen*	*High sleep, increasing outdoor time, low screen*	*High screen, increasing outdoor time, high sleep*
***Maternal knowledge, beliefs, attitudes and expectations***^***a***^				
PA knowledge	1.00	0.51 (0.23, 1.12)	0.81 (0.40, 1.63)	0.74 (0.30, 1.85)
Views of physically active children	1.00	**0.42 (0.23, 0.76)**	**0.57 (0.33, 0.96)**	**0.35 (0.16, 0.77)**
PA optimism	1.00	**0.56 (0.33, 0.97)**	0.67 (0.41, 1.09)	0.61 (0.28, 1.34)
Self-efficacy for promoting PA	1.00	0.56 (0.31, 1.04)	0.76 (0.45, 1.30)	**0.43 (0.24, 0.78)**
Future expectations for children’s PA & ST	1.00	**0.61 (0.39, 0.95)**	0.72 (0.48, 1.08)	0.74 (0.43, 1.30)
Perceptions of floor play safety	1.00	**2.73 (1.86, 4.02)**	1.32 (0.94, 1.87)	1.49 (0.78, 2.86)
ST knowledge	1.00	**0.45 (0.25, 0.80)**	**0.52 (0.34, 0.80)**	**0.07 (0.03, 0.19)**
ST use for practical reasons	1.00	**0.32 (0.18, 0.55)**	**0.46 (0.28, 0.75)**	**0.10 (0.05, 0.21)**
Self-efficacy for limiting ST	1.00	**0.49 (0.32, 0.75)**	**0.55 (0.37, 0.82)**	**0.46 (0.28, 0.76)**
***Maternal behaviours***				
MVPA (hours/day)	1.00	**0.77 (0.60, 1.00)**	**0.60 (0.47, 0.76)**	0.73 (0.49, 1.09)
ST (hours/day)	1.00	1.30 (0.56, 3.02)	0.83 (0.39, 1.74)	**4.36 (1.83, 10.41)**
Good sleep quality^b^	1.00	0.68 (0.38, 1.24)	1.28 (0.74, 2.24)	0.90 (0.40, 2.04)

Notes: ^a^ Higher score indicates maternal beliefs, attitudes and expectations are in line with evidence/recommendations; ^b^ categorical variable (reference category = bad sleep quality); analyses adjusted for child sex and baseline age, intervention allocation, and clustering by first-time parent group; boldface denotes statistical significance (*p* < 0.05)

Abbreviations: *CI* Confidence interval, *MVPA* Moderate- to vigorous-intensity physical activity, *OR* Odds ratio, *PA* Physical activity, *ST* Screen time

To compare differences in maternal predictors between the other three groups, we also ran multinomial logistic regression models with the *‘unstable sleep, increasing outdoor time, low screen’* and *‘high sleep, increasing outdoor time, low screen’* groups as the reference categories (see Additional file 3; Tables S3 and S4). Perceptions of floor play were associated with 52% decreased odds of children being in the *‘high sleep, increasing outdoor time, low screen’* group, while screen time knowledge and screen time use for practical reasons were associated with 85% and 68% decreased odds, respectively, of children being in the *‘high screen, increasing outdoor time, high sleep’* group compared to the *‘unstable sleep, increasing outdoor time, low screen’* group. Maternal MVPA was associated with 23% lower odds, and maternal sleep quality with 88% higher odds, of children being in *‘high sleep, increasing outdoor time, low screen’* compared to the *‘unstable sleep, increasing outdoor time, low screen’* group. Children had 235% higher odds of being in the *‘high screen, increasing outdoor time, high sleep’* compared to the *‘unstable sleep, increasing outdoor time, low screen’* group with each additional hour of maternal screen time. Finally, when comparing the *‘high sleep, increasing outdoor time, low screen’* and *‘high screen, increasing outdoor time, high sleep’* groups, self-efficacy for promoting physical activity, screen time knowledge, and screen time use for practical reasons were associated with 43%, 87% and 78% decreased odds, respectively, while maternal screen time was associated with 427% increased odds, of children being in the *‘high screen, increasing outdoor time, high sleep’* group.

### Sensitivity results

Utilising the same model fit characteristics as the main trajectory analyses, the following four groups were identified (see Additional file 4; Figure S1): Group 1, characterised by increasing physical activity, unstable/high screen time and low sleep, comprised 10.6% of the sample. Group 2, characterised by high physical activity, low screen time, and low sleep, comprised 16.6% of the sample. The majority of the sample (64.5%) belonged to Group 3, characterised by high physical activity, low screen time, and high sleep. Finally, Group 4, comprising 8.2% of the sample, was characterised by low physical activity, high/increasing screen time, and high sleep. Multinomial regression results showed similar results to those in the main analyses (see Additional file 4; Table S5). Using Group 3 as the referent group, screen time knowledge, screen time use for practical reasons, and maternal screen time were associated with decreased odds of being in both Groups 1 and 4 (both of which had generally higher levels of screen time).

## Discussion

In this study, we modelled concurrent trajectories of outdoor time, screen time and sleep across the early childhood period (from 4 to 60 months). Consistent with previous evidence, both outdoor time and screen time tended to increase with age [40], while sleep duration decreased [10]. However, our concurrent trajectory analyses identified four discrete patterns of change over time within individuals. Almost half the sample belonged to the *‘high sleep, increasing outdoor time, low screen group’*, one-quarter of the sample belonged to either the *‘unstable sleep, increasing outdoor time, low screen’* or *‘high outdoor time, low screen, high sleep’* groups, and just 10% were categorized in the *‘high screen, increasing outdoor time, high sleep’* group. There were a number of maternal factors that appeared to be associated with group membership.

The groups identified in the current study were mainly characterized by differences in just one of the three behaviours, i.e., there were no distinct patterns in behaviours grouping together. For example, the *‘unstable sleep, increasing outdoor time, low screen’* group was characterised by unstable sleep with average trajectories for outdoor time and screen time, while the *‘high outdoor time, low screen, high sleep’* group was characterised by higher-than-average outdoor time and average trajectories for screen time and sleep. This is not dissimilar to previous evidence that found three distinct joint-trajectories of physical activity and screen time from birth to 5 years, similarly characterized by a single stand out behaviour: a *‘low activity-low screen’*, *‘increasing activity-low screen’*, and *‘low activity-increasing screen’*group [15]. Collectively, these findings suggest that there may not be a strong interplay between movement behaviours in early childhood; i.e., children tend to be ‘average’ overall but have a single defining behaviour. When designing interventions, it may be important to identify these defining behaviours (i.e., using trajectories) within a cohort and target changes in that specific behaviour.

The differences in outdoor time and screen time between the groups with the lowest and highest levels of the respective behaviours were magnified over time. Outdoor time differed between groups by 30 min at age 4 months, increasing to almost 2 h at 60 months. Similarly, the difference in screen time between groups at age 4 months was around 1.5 h/day, increasing to more than 2.5 h/day at 60 months. These findings underscore the need for early identification of children at risk of unhealthy trajectories of these behaviours, and subsequent early intervention to promote healthy behaviours across the early childhood period. Conversely, sleep stabilized over time, with the difference between the groups with the lowest and highest levels of sleep decreasing from around 4 h/day at age 4 months to less than 1 h/day at 60 months. This finding likely reflects the greater stability of sleep by school starting age, compared to infants where sleep issues are prevalent [41].

The maternal factors that predicted membership in different concurrent trajectories of movement behaviours may be important to consider in the planning of interventions to promote healthy movement behaviours. Higher maternal self-efficacy for limiting screen time and promoting physical activity was predictive of children being in the healthiest group, characterized by high outdoor time. Existing evidence suggests that parental self-efficacy appears to be generalizable across movement behaviors [21, 42], and has also been shown to track over time [43], suggesting that targeting parents’ self-efficacy early, i.e., prior to their children’s first exposure to screens, may be important.

Higher maternal screen time knowledge and lower screen time use for practical reasons were associated with reduced odds of children being in the groups characterized by unstable sleep and high screen time. Additionally, maternal television viewing was strongly associated with increased odds of children being in the group characterized by high screen time. These findings are consistent with previous evidence showing that parental attitudes regarding their child’s screen time (encompassing knowledge and use) and parent’s own screen time are related to children’s screen time [44]. Other potentially important maternal factors identified in this study were maternal views of physically active children and mothers’ own MVPA. Together, these findings suggest that mothers who have a good understanding and value the importance of moving more and minimizing screen time are likely to be doing this more themselves, and promoting this more in their children. Targeting mothers’ modelling of movement behaviours, in addition to their knowledge and beliefs, may be important to promote healthy behaviours from a young age.

Mothers’ sleep quality was associated with higher odds of children being in the group characterized by high sleep compared to the group characterized by unstable sleep. Previous research has found that a number of factors are associated with infant sleep, including co-sleeping, being nursed to sleep, longer sleep latency (i.e., taking longer to fall asleep), and longer and more frequent night-waking [41], and it is likely that many of these factors also affect maternal sleep quality. In the current study, mothers reported their sleep quality at baseline (child age 4 months), so it is possible that there were already bi-directional associations between sleep quality and their infants’ sleep at this age, where the largest difference (of over 4 h) was observed between these groups characterized by high vs unstable sleep.

There are some limitations of the present study. The key limitation is the use of outdoor time as a proxy for physical activity at each time point. Mothers were not asked to report whether this time outdoors was active or sedentary. Evidence from 2-year-old children attending childcare suggests that almost 70% of their time outdoors is active, with 21% of total time outdoors being MVPA [19]. Preliminary evidence suggests that around 57% of infants’ and toddlers’ (ranging from 6 weeks to 36 months of age) unstructured outdoor time in childcare is spent active [20]. Although this study included a relatively small sample (*n* = 49) in a childcare setting, results provide initial evidence that outdoor time can be active time in very young children. It is likely that a portion of the reported time outdoors in the current study was spent sedentary for children who were mobile. At the earlier time points (particularly at baseline when children were 4 months old), it is likely that a large portion of time outdoors was spent in a pram/stroller and this time may not accurately represent physical activity per se. However, ‘physical activity’ in the first 6 months of life comprises small movements such as reaching and grasping objects, turning the head toward a stimulus, and movement of the arms and legs [45], all of which are difficult to measure (either objectively or via parent report). In children aged 2 years and over, time outdoors is positively associated with habitual physical activity [34], and is therefore often used as a proxy for physical activity [32, 33]. As physical activity has been shown to track through early childhood [11], time outdoors at a very young age may be predictive of time outdoors in future years, and therefore predictive of future physical activity levels. Given this, and that the operationalization of physical activity guideline compliance differs across the early childhood period (i.e., tummy time for infants and total physical activity with a focus on intensity [i.e., MVPA] for toddlers and preschoolers), we decided to use outdoor time for our trajectory analyses as a common indicator of physical activity across the five time points. Results from our sensitivity analyses provide confidence in the findings.

Secondly, mothers in our sample were highly educated and recruited from Metropolitan areas within 60 km of Deakin University’s Burwood campus, which may preclude generalizability to the wider population. Thirdly, data for the main analyses (i.e., children’s outdoor time, screen time, sleep, and parental predictors) were all parent-reported. As such, results may be subject to reporting biases, whereby mothers may have reported their own and their child’s behaviours to be more socially desirable. Fourthly, data were drawn from a randomised controlled trial. It is important to acknowledge that use of data from a trial may have affected the children’s behaviours, and results should be interpreted with this in mind. However, there was no difference in sleep duration between the intervention and control groups, no intervention effect was observed for physical activity, the intervention effect for screen time was attenuated at follow-up, and intervention allocation was included as a covariate in regression analyses. Finally, data were collected in 2008–2013; newer screen technologies such as smartphones and tablet computers were not as ubiquitous then and hence time spent using these devices was not measured. As such, screen time may have been underestimated, particularly at the later time points, which is especially concerning for the group with the highest screen time. Despite these limitations, this study has several strengths. This is the first study to examine concurrent trajectories of outdoor time, screen time and sleep across the early childhood period. We had longitudinal data on outdoor time, screen time and sleep at five time points across the early childhood period in a relatively large sample. In addition, the use of the rigorous analytical technique, GBTM, to examine concurrent trajectories of outdoor time, screen time and sleep in this population is highly novel. A particular strength of this technique is its ability to allow inclusion of participants without complete data, which often hinders analyses in longitudinal samples with multiple time points.

## Conclusions

Four distinct trajectories of outdoor time, screen time and sleep duration across the early childhood period were identified, with approximately one quarter of the sample belonging to the group that was seemingly the healthiest; characterized by high outdoor time along with low levels of screen time and healthy sleep duration. A number of maternal factors were identified that were supportive of children being in this group, with positive views of physically active children, screen time knowledge, limited use of screen time use for practical reasons, self-efficacy for limiting screen time, and maternal MVPA levels the most consistent predictors. Conversations around these factors to build parental knowledge, skills and confidence may be important to incorporate into clinical practice. Additionally, future interventions and public policy may benefit from targeting these factors to support healthy movement behaviours from a young age.

## Supplementary Information

**Additional file 1.** 

**Additional file 2.** 

**Additional file 3.** 

**Additional file 4.**

## Data Availability

The datasets analysed for the current study are not publicly available due to ethical restrictions related to the consent given by participants at the time of study commencement. An ethically compliant dataset may be made available by the corresponding author on reasonable request and upon approval by the Deakin University Human Research Ethics Committee.

## Abbreviations

BMI: Body mass index
GBTM: Group-based trajectory modelling
MVPA: Moderate- to vigorous-intensity physical activity
