# Supplementary material for: Outdoor time, screen time and sleep reported across early childhood: concurrent trajectories and maternal predictors
Source: Int J Behav Nutr Phys Act. 2022 Dec 29;19:160. doi: 10.1186/s12966-022-01386-x (PMC9798690; doi:10.1186/s12966-022-01386-x)
Supplement: Supplementary file 1 — Additional file 1. [file 12966_2022_1386_MOESM1_ESM.docx]

Table S1. Model fit characteristics

| Trajectory group | Group membership | BIC (n=6379) | BIC (n=528) | AIC |
| --- | --- | --- | --- | --- |
| 2-group model |  | -10413.83 | -10386.42 | -10339.46 |
| 1 | 86.3 |  |  |  |
| 2 | 13.7 |  |  |  |
| 3-group model |  | -10305.29 | -10265.43 | -10197.12 |
| 1 | 25.5 |  |  |  |
| 2 | 63.6 |  |  |  |
| 3 | 10.9 |  |  |  |
| 4-group model |  | -10234.74 | -10182.42 | -10092.77 |
| 1 | 21.7 |  |  |  |
| 2 | 23.9 |  |  |  |
| 3 | 44.5 |  |  |  |
| 4 | 9.9 |  |  |  |
| 5-group model* |  | -10332.08 | -10267.30 | -10156.30 |
| 1 | 5.6 |  |  |  |
| 2 | 57.3 |  |  |  |
| 3 | 29.5 |  |  |  |
| 4 | 7.6 |  |  |  |
| 5 | 0.0 |  |  |  |

* variance matrix was nonsymmetric or highly singular so maximum likelihood estimates were not computed
