# Supplementary material for: Outdoor time, screen time and sleep reported across early childhood: concurrent trajectories and maternal predictors
Source: Int J Behav Nutr Phys Act. 2022 Dec 29;19:160. doi: 10.1186/s12966-022-01386-x (PMC9798690; doi:10.1186/s12966-022-01386-x)
Supplement: Supplementary file 2 — Additional file 2. [file 12966_2022_1386_MOESM2_ESM.docx]

Table S2. Descriptive characteristics of time in movement behaviors

|  | Total sample | | | Group 1 (21.7%):  *Unstable sleep, increasing outdoor time, low screen* | | | Group 2 (23.9%):  *High outdoor time, low screen, high sleep* | | | Group 3 (44.5%):  *High sleep, increasing outdoor time, low screen* | | | Group 4 (9.9%):  *High screen, increasing outdoor time, high sleep* | | |
| --- | --- | --- | --- | --- | --- | --- | --- | --- | --- | --- | --- | --- | --- | --- | --- |
|  | Mean (SD) h/day | | | Mean (SD) h/day | | | Mean (SD) h/day | | | Mean (SD) h/day | | | Mean (SD) h/day | | |
| Age (mo) | Outdoor time | Screen time | Sleep | Outdoor time | Screen time | Sleep | Outdoor time | Screen time | Sleep | Outdoor time | Screen time | Sleep | Outdoor time | Screen time | Sleep |
| 4 | 0.68  (0.73) | 0.61  (0.96) | 14.00  (2.32) | 0.61  (0.60) | 0.55  (0.78) | 11.08  (1.58) | 1.04  (1.04) | 0.37  (0.50) | 14.28  (1.71) | 0.52  (0.60) | 0.48  (0.70) | 15.18  (1.71) | 0.73  (0.85) | 2.00  (1.81) | 14.23  (1.98) |
| 9 | 1.00  (0.83) | 0.59  (0.91) | 13.73  (1.51) | 0.87  (0.70) | 0.56  (0.75) | 12.35  (1.54) | 1.52  (0.92) | 0.36  (0.48) | 13.59  (1.32) | 0.77  (0.63) | 0.46  (0.59) | 14.39  (1.21) | 1.10  (1.05) | 1.85  (1.87) | 13.78  (1.18) |
| 19 | 1.77  (1.21) | 0.89  (0.99) | 13.31  (1.24) | 1.54  (1.10) | 0.82  (0.74) | 12.71  (1.38) | 2.80  (1.41) | 0.54  (0.54) | 13.17  (1.15) | 1.35  (0.83) | 0.72  (0.69) | 13.61  (1.10) | 1.69  (0.87) | 2.63  (1.57) | 13.38  (1.34) |
| 42 | 2.87  (1.44) | 1.88  (1.36) | 11.51  (1.02) | 2.52  (1.27) | 2.09  (1.46) | 11.09  (1.05) | 4.28  (1.13) | 1.50  (1.06) | 11.47  (0.88) | 2.22  (0.99) | 1.57  (0.82) | 11.71  (0.98) | 3.20  (1.70) | 3.89  (2.02) | 11.54  (1.22) |
| 60 | 2.86  (1.36) | 2.15  (1.36) | 10.89  (0.82) | 2.29  (1.10) | 1.97  (1.06) | 10.36  (0.82) | 4.25  (1.12) | 1.64  (1.03) | 10.99  (0.65) | 2.35  (1.04) | 2.06  (1.08) | 11.12  (0.78) | 3.00  (1.23) | 4.35  (1.90) | 10.61  (0.86) |

Abbreviations: h, hour; mo, months; SD, standard deviation
