# Supplementary material for: Outdoor time, screen time and sleep reported across early childhood: concurrent trajectories and maternal predictors
Source: Int J Behav Nutr Phys Act. 2022 Dec 29;19:160. doi: 10.1186/s12966-022-01386-x (PMC9798690; doi:10.1186/s12966-022-01386-x)
Supplement: Supplementary file 3 — Additional file 3. [file 12966_2022_1386_MOESM3_ESM.docx]

Table S3. Associations of maternal factors with multi-trajectories of movement behaviors

|  | OR (95% CI) | | |
| --- | --- | --- | --- |
|  | *Unstable sleep, increasing outdoor time, low screen* (referent group) | *High sleep, increasing outdoor time, low screen* | *High screen, increasing outdoor time, high sleep* |
| ***Maternal knowledge, beliefs, attitudes and expectations^a^*** |  |  |  |
| PA knowledge | 1.00 | 1.59 (0.81, 3.12) | 1.45 (0.53, 4.00) |
| Views of physically active children | 1.00 | 1.34 (0.82, 2.21) | 0.83 (0.35, 1.93) |
| PA optimism | 1.00 | 1.18 (0.75, 1.87) | 1.08 (0.49, 2.36) |
| Self-efficacy for promoting PA | 1.00 | 1.35 (0.81, 2.23) | 0.76 (0.41, 1.40) |
| Future expectations for children’s PA & ST | 1.00 | 1.18 (0.79, 1.77) | 1.22 (0.70, 2.13) |
| Floor play concerns | 1.00 | **0.48 (0.32, 0.72)** | 0.55 (0.26, 1.14) |
| ST knowledge | 1.00 | 1.15 (0.69, 1.93) | **0.15 (0.05, 0.45)** |
| ST use for practical reasons | 1.00 | 1.45 (0.96, 2.19) | **0.32 (0.15, 0.69)** |
| Self-efficacy for limiting ST | 1.00 | 1.12 (0.82, 1.54) | 0.94 (0.56, 1.57) |
| ***Maternal behaviours*** |  |  |  |
| MVPA (hours/day) | 1.00 | **0.77 (0.60, 1.00)** | 0.94 (0.61, 1.45) |
| ST (hours/day) | 1.00 | 0.63 (0.31, 1.28) | **3.35 (1.81, 6.18)** |
| Good sleep quality^b^ | 1.00 | **1.88 (1.04, 3.37)** | 1.31 (0.58, 2.94) |

Notes: ^a^ Higher score indicates maternal beliefs, attitudes and expectations are in line with evidence/recommendations; ^b^ categorical variable (reference category = bad sleep quality); analyses adjusted for child sex and baseline age, intervention allocation, and clustering by first-time parent group; boldface denotes statistical significance (p<0.05)

Abbreviations: CI, confidence interval; MVPA, moderate- to vigorous-intensity physical activity; OR, odds ratio; PA, physical activity; ST, screen time

Table S4. Associations of maternal factors with multi-trajectories of movement behaviors

|  | OR (95% CI) | |
| --- | --- | --- |
|  | *High sleep, increasing outdoor time, low screen*  (referent group) | *High screen, increasing outdoor time, high sleep* |
| ***Maternal knowledge, beliefs, attitudes and expectations^a^*** |  |  |
| PA knowledge | 1.00 | 0.91 (0.42, 2.00) |
| Views of physically active children | 1.00 | 0.62 (0.29, 1.33) |
| PA optimism | 1.00 | 0.91 (0.43, 1.92) |
| Self-efficacy for promoting PA | 1.00 | **0.57 (0.35, 0.92)** |
| Future expectations for children’s PA & ST | 1.00 | 1.03 (0.59, 1.82) |
| Floor play concerns | 1.00 | 1.13 (0.57, 2.22) |
| ST knowledge | 1.00 | **0.13 (0.05, 0.38)** |
| ST use for practical reasons | 1.00 | **0.22 (0.12, 0.42)** |
| Self-efficacy for limiting ST | 1.00 | 0.84 (0.51, 1.37) |
| ***Maternal behaviours*** |  |  |
| MVPA (hours/day) | 1.00 | 1.22 (0.80, 1.87) |
| ST (hours/day) | 1.00 | **5.27 (2.78, 10.00)** |
| Good sleep quality^b^ | 1.00 | 0.70 (0.33, 1.51) |

Notes: ^a^ Higher score indicates maternal beliefs, attitudes and expectations are in line with evidence/recommendations; ^b^ categorical variable (reference category = bad sleep quality); analyses adjusted for child sex and baseline age, intervention allocation, and clustering by first-time parent group; boldface denotes statistical significance (p<0.05)

Abbreviations: CI, confidence interval; MVPA, moderate- to vigorous-intensity physical activity; OR, odds ratio; PA, physical activity; ST, screen time
