# Supplementary material for: Outdoor time, screen time and sleep reported across early childhood: concurrent trajectories and maternal predictors
Source: Int J Behav Nutr Phys Act. 2022 Dec 29;19:160. doi: 10.1186/s12966-022-01386-x (PMC9798690; doi:10.1186/s12966-022-01386-x)
Supplement: Supplementary file 4 — Additional file 4. [file 12966_2022_1386_MOESM4_ESM.docx]

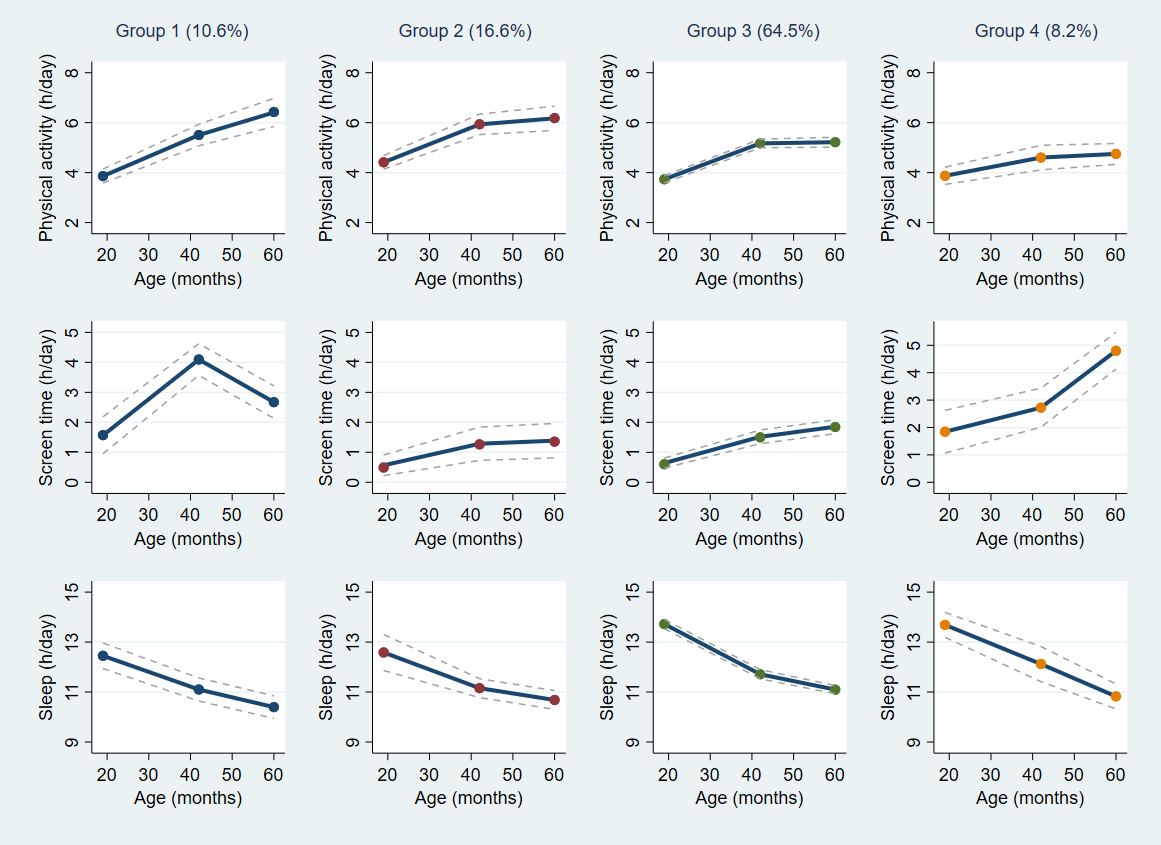


**Figure S1.** Sensitivity results for concurrent trajectories of physical activity, screen time and sleep

Note: Physical activity refers to accelerometer-derived total physical activity (i.e., light-, moderate- and vigorous-intensity physical activity)

**Table S5.** Sensitivity results for associations of maternal factors with concurrent trajectories of movement behaviours

|  | OR (95% CI) | | | |
| --- | --- | --- | --- | --- |
|  | *Group 3* (referent group) | *Group 1* | *Group 2* | *Group 4* |
| ***Maternal knowledge,*** ***beliefs, attitudes and expectations^a^*** |  |  |  |  |
| PA knowledge | 1.00 | 0.67 (0.24, 1.90) | 0.67 (0.31, 1.41) | 0.51 (0.17, 1.53) |
| Views of physically active children | 1.00 | 0.62 (0.31, 1.26) | 0.81 (0.43, 1.53) | 0.89 (0.42, 1.91) |
| PA optimism | 1.00 | 0.69 (0.30, 1.52) | 1.18 (0.63, 2.22) | 0.86 (0.42, 1.75) |
| Self-efficacy for promoting PA | 1.00 | 0.68 (0.40, 1.47) | 0.87 (0.51, 1.47) | 0.76 (0.42, 1.39) |
| Future expectations for children’s PA & ST | 1.00 | 0.78 (0.41, 1.48) | 0.85 (0.53, 1.35) | 0.84 (0.52, 1.35) |
| Perceptions of floor play safety | 1.00 | 1.01 (0.58, 1.76) | 0.95 (0.56, 1.63) | 1.36 (0.76, 2.42) |
| ST knowledge | 1.00 | **0.32 (0.14, 0.72)** | 0.81 (0.50, 1.32) | **0.18 (0.07, 0.48)** |
| ST use for practical reasons | 1.00 | **0.41 (0.21, 0.81)** | 1.23 (0.67, 2.23) | **0.36 (0.15, 0.86)** |
| Self-efficacy for limiting ST | 1.00 | 0.63 (0.39, 1.01) | 1.00 (0.64, 1.56) | 0.79 (0.42, 1.46) |
| ***Maternal behaviours*** |  |  |  |  |
| MVPA (hours/day) | 1.00 | 1.00 (0.64, 1.58) | 1.05 (0.76, 1.44) | 1.12 (0.77, 1.64) |
| ST (hours/day) | 1.00 | **2.89 (1.32, 6.32)** | 0.92 (0.37, 2.61) | **3.72 (1.90, 7.29)** |
| Good sleep quality^b^ | 1.00 | 0.88 (0.41, 1.87) | 0.87 (0.50, 1.54) | 0.97 (0.40, 2.35) |

Notes: ^a^ Higher score indicates maternal beliefs, attitudes and expectations are in line with evidence/recommendations; ^b^ categorical variable (reference category = bad sleep quality); analyses adjusted for child sex and baseline age, intervention allocation, and clustering by first-time parent group; boldface denotes statistical significance (p<0.05)

Abbreviations: CI, confidence interval; MVPA, moderate- to vigorous-intensity physical activity; OR, odds ratio; PA, physical activity; ST, screen time
